# Retraction: Juan Hu, Qiang Li, Yingxin Huang, Qilin Zhang, Daowei Zhou, Distributions of trace elements with long‐term grazing exclusion in a semi‐arid grassland of inner Mongolia. *Ecol Evol*. 2022; 12: e9237. https://doi.org/10.1002/ece3.9237

**DOI:** 10.1002/ece3.9478

**Published:** 2022-11-28

The above article, published online on 23 August 2022 in Wiley Online Library (wileyonlinelibrary.com), has been retracted by agreement between the authors, the journal's Editors in Chief (Allen Moore, Andrew Beckerman, Marcus Lashley, Chris Foote, Gareth Jenkins, and Zoe Ma), and John Wiley and Sons Ltd. The retraction has been agreed due to the use of an incorrect conversion ratio to calculate community root biomass from the field‐collected original data, which has led to a change in some key findings.

